# Unveiling the landscape of pathomics in personalized immunotherapy for lung cancer: a bibliometric analysis

**DOI:** 10.3389/fonc.2024.1432212

**Published:** 2024-07-08

**Authors:** Lei Yuan, Zhiming Shen, Yibo Shan, Jianwei Zhu, Qi Wang, Yi Lu, Hongcan Shi

**Affiliations:** ^1^ Department of Thoracic Surgery, Northern Jiangsu People’s Hospital Affiliated to Yangzhou University, Yangzhou, China; ^2^ Institute of Translational Medicine, Medical College, Yangzhou University, Yangzhou, China; ^3^ Jiangsu Key Laboratory of Integrated Traditional Chinese and Western Medicine for Prevention and Treatment of Senile Diseases, Yangzhou University, Yangzhou, China

**Keywords:** lung cancer, pathomics, artificial intelligence, deep learning, tumor microenvironment, immunotherapy

## Abstract

**Background:**

Pathomics has emerged as a promising biomarker that could facilitate personalized immunotherapy in lung cancer. It is essential to elucidate the global research trends and emerging prospects in this domain.

**Methods:**

The annual distribution, journals, authors, countries, institutions, and keywords of articles published between 2018 and 2023 were visualized and analyzed using CiteSpace and other bibliometric tools.

**Results:**

A total of 109 relevant articles or reviews were included, demonstrating an overall upward trend; The terms “deep learning”, “tumor microenvironment”, “biomarkers”, “image analysis”, “immunotherapy”, and “survival prediction”, etc. are hot keywords in this field.

**Conclusion:**

In future research endeavors, advanced methodologies involving artificial intelligence and pathomics will be deployed for the digital analysis of tumor tissues and the tumor microenvironment in lung cancer patients, leveraging histopathological tissue sections. Through the integration of comprehensive multi-omics data, this strategy aims to enhance the depth of assessment, characterization, and understanding of the tumor microenvironment, thereby elucidating a broader spectrum of tumor features. Consequently, the development of a multimodal fusion model will ensue, enabling precise evaluation of personalized immunotherapy efficacy and prognosis for lung cancer patients, potentially establishing a pivotal frontier in this domain of investigation.

## 1 Introduction

Lung cancer remains one of the most prevalent malignancies and represents the foremost cause of cancer-related mortality worldwide (1, 2), the majority of lung cancers (80–90%) manifest as non-small cell lung cancer (NSCLC), often diagnosed at an advanced stage (65%), potentially with concurrent local or distant metastasis (3). Recent advances in immunotherapy, particularly the use of immune checkpoint inhibitors (ICIs), have shown promising outcomes in enhancing the prognosis of lung cancer patients (4). Nevertheless, not all patients experience the benefits of immunotherapy, highlighting the need for additional research into predictive biomarkers of immune response. These biomarkers, which may include substances, structures, or products of processes within the body, have the potential to facilitate personalized immunotherapy by enabling the monitoring of immune reactions.

Each lung cancer patient undergoes histopathological diagnosis, involving the preparation of biopsy tissues into pathological slides for examination. The traditional preservation method of using wax embedding techniques for pathological slides can now be digitized through computerization, archiving them as digital pathology images. This technological advancement serves as a foundation for applying big data analytics to digital pathology images. Consequently, the field of pathomics has emerged (5). Pathomics entails applying machine learning techniques to extract large-scale, objectively quantifiable, and readily analyzable datasets from digitally scanned pathological tissue images. Consistent with the pathological diagnostic requirements of diseases, morphological features, including size and shape of pathological images, along with multi-dimensional subtle features reflecting potential biological characteristics such as texture features and edge gradient features, are extracted. These features can be utilized for quantitative disease screening, diagnosis, prognosis prediction, and other applications (6).

In this study, CiteSpace (7) was utilized for the inaugural analysis of hotspots and trends in the application of pathomics in lung cancer. The objective is to provide valuable insights for scholars involved in research within this domain.

## 2 Materials and methods

### 2.1 Data collection

Web of Science Core Collection (WoSCC) database was chosen as the literature retrieval platform. The retrieval period spanned from 2018 to 2023, with the final search conducted on October 20, 2023. Subject terms were exclusively employed as the search method, and the search formula was: TS= (“Pathomics” OR “Pathomics” OR “Digital Pathology” OR “Whole-slide Imaging” OR “Whole Slide Imaging” OR “Computational Pathology”) AND TS=(“Lung Cancer” OR “Pulmonary Cancer” OR “Carcinoma of Lung” OR “Pulmonary Carcinoma” OR “Cancer of Lung” OR “Bronchogenic Carcinoma” OR “Bronchogenic” OR “Cancer of the Lung” OR “NSCLC” OR “SLC”), document type: Articles or Review Articles; a total of 109 documents were retrieved.

### 2.2 Statistical methods

Export the complete records and referenced bibliographies of the 109 documents retrieved from WoSCC in Text format, comprising 85 articles and 24 reviews. Conduct a comprehensive analysis of the literature using CiteSpace 6.2.R4 (64-bit) Basic, focusing on the country, institution, authorship, keywords, and cited references. The bibliometric online analysis platform, developed by the National Science Library of the Chinese Academy of Sciences, was employed to conduct a visual analysis of historical keywords and national collaborations.

## 3 Results

### 3.1 Annual publication volume in WoSCC

**Figure 1:**
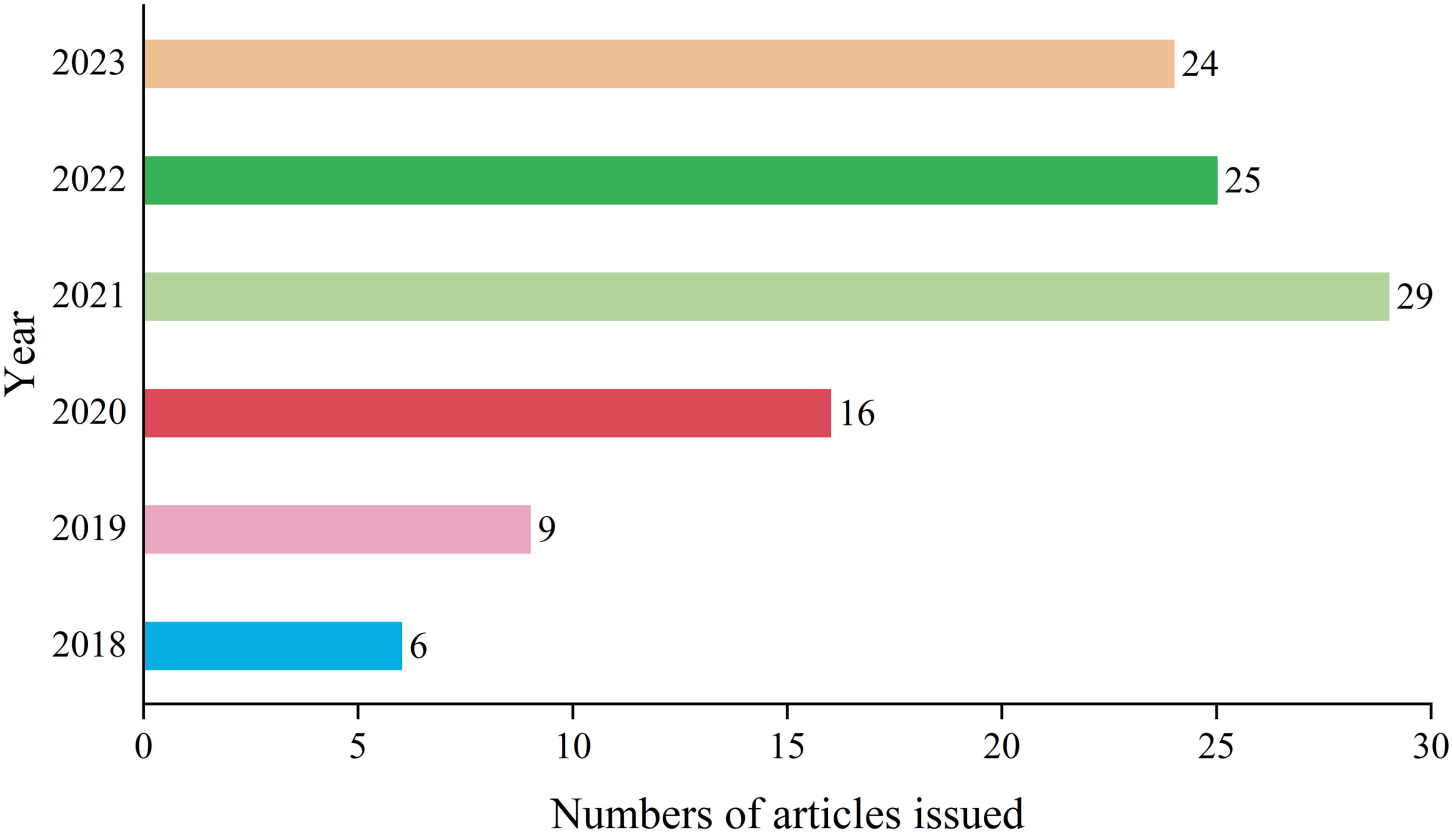
Annual analysis of the number of articles issued.

A total of 109 matching documents were retrieved, and the overall publication output exhibited a general upward trend, especially reaching a contribution rate of 26.61% in 2021 (**Figure 1**). The annual average publication output is approximately 21.8 articles. The results indicate a gradual increase in the attention to pathomics research in the context of lung cancer.

### 3.2 Distribution of source journals

The literature selected from the 109 studies on pathomics in the management of lung cancer has been indexed by 146 journals. For the top 10 journals in terms of publication output, detailed information on Journal Citation Reports (JCR) category, publication quantity, impact factor (IF), and their respective contribution percentages is provided in **Table 1**.

**Table 1:** Top 10 journals in terms of publication volume.

Journal Titles	JCR	Number	IF	Rate%
CANCERS	Q1	11	5.2	10.092
MODERN PATHOLOGY	Q1	6	7.5	5.505
EBIOMEDICINE	Q1	3	11.1	2.752
FRONTIERS IN ONCOLOGY	Q2	3	4.7	2.752
HISTOPATHOLOGY	Q1	3	6.4	2.752
IEEE ACCESS	Q2	3	3.9	2.752
MEDICAL IMAGE ANALYSIS	Q1	3	10.9	2.752
BIOINFORMATICS	Q1	2	5.8	1.835
COMPUTERS IN BIOLOGY AND MEDICINE	Q1	2	7.7	1.835
IEEE TRANSACTIONS ON MEDICAL IMAGING	Q1	2	10.6	1.835

### 3.3 Visualization of collaborations between countries and institutions

**Figure 2:**
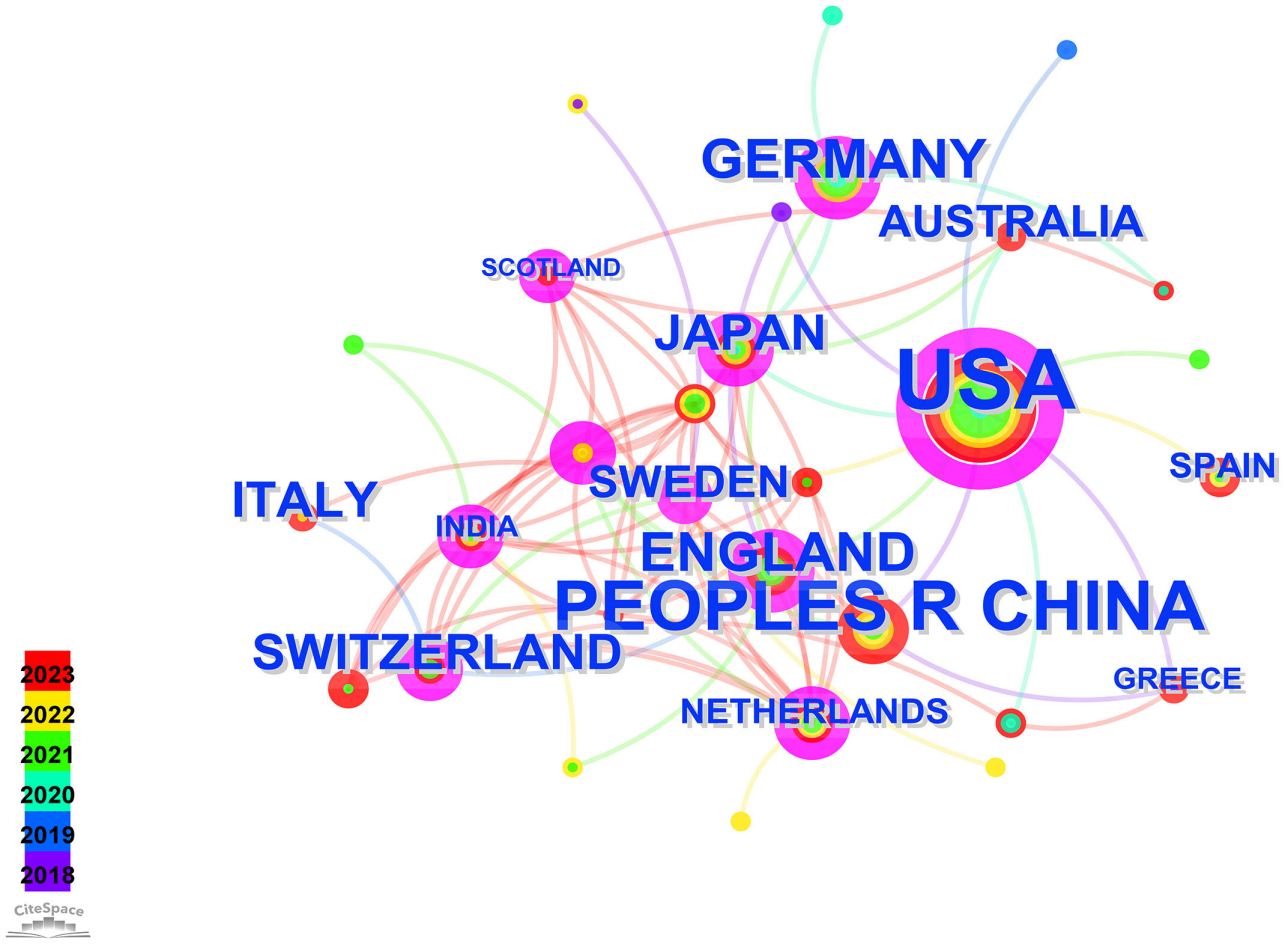
Visual map of countries.

Running the CiteSpace software for country analysis resulted in a knowledge graph with 35 nodes and 80 edges (**Figure 2**). Each circular node represents a country, with the size indicating the quantity of publications from that country. The connections between nodes represent collaborative relationships between countries, with the thickness of the connections reflecting the degree of collaboration. Different colors of nodes represent different time periods (8), the size of the purple circles reflects the centrality values indicating the influence of each country.

**Figure 3:**
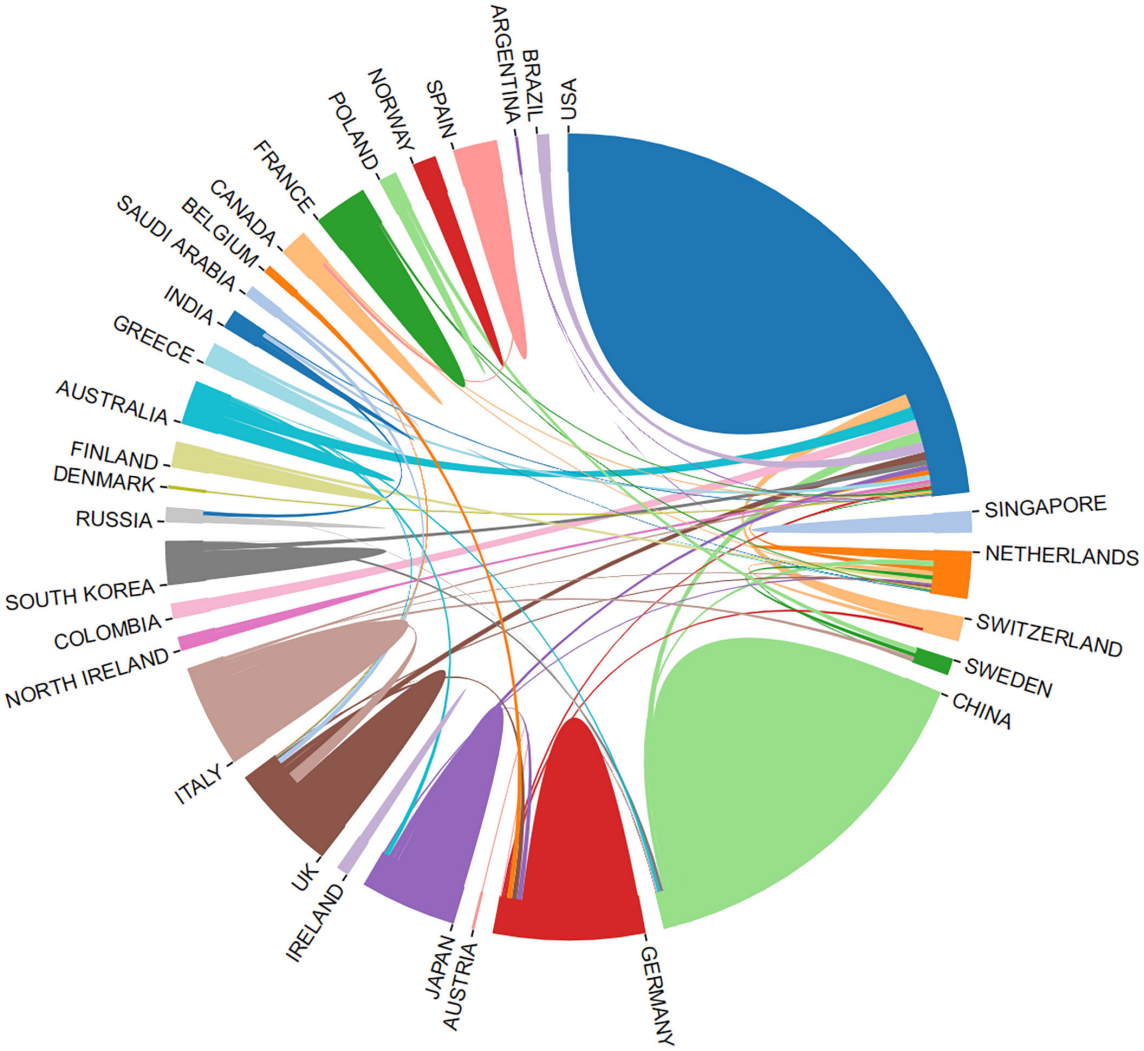
Proportion of national contribution.

Leveraging the bibliometric online analysis platform, **Figure 3** depicts the contributions of different countries in the field. Distinctly colored blocks represent the proportional contribution of each country. **Table 2** presents the top 5 institutions in terms of publication output.

**Table 2:** Top 5 institutions in terms of publication volume.

Rank	Number	Institution	Country
1	8	CASE WESTERN RESERVE UNIVERSITY	USA
2	8	UNIVERSITY OF TEXAS SYSTEM	USA
3	6	LOUIS STOKES CLEVELAND VETERANS AFFAIRS MEDICAL CENTER	USA
4	6	UNIVERSITY OF TEXAS SOUTHWESTERN MEDICAL CENTER DALLAS	USA
5	6	US DEPARTMENT OF VETERANS AFFAIRS	USA

### 3.4 Visualization of author collaborations

**Figure 4:**
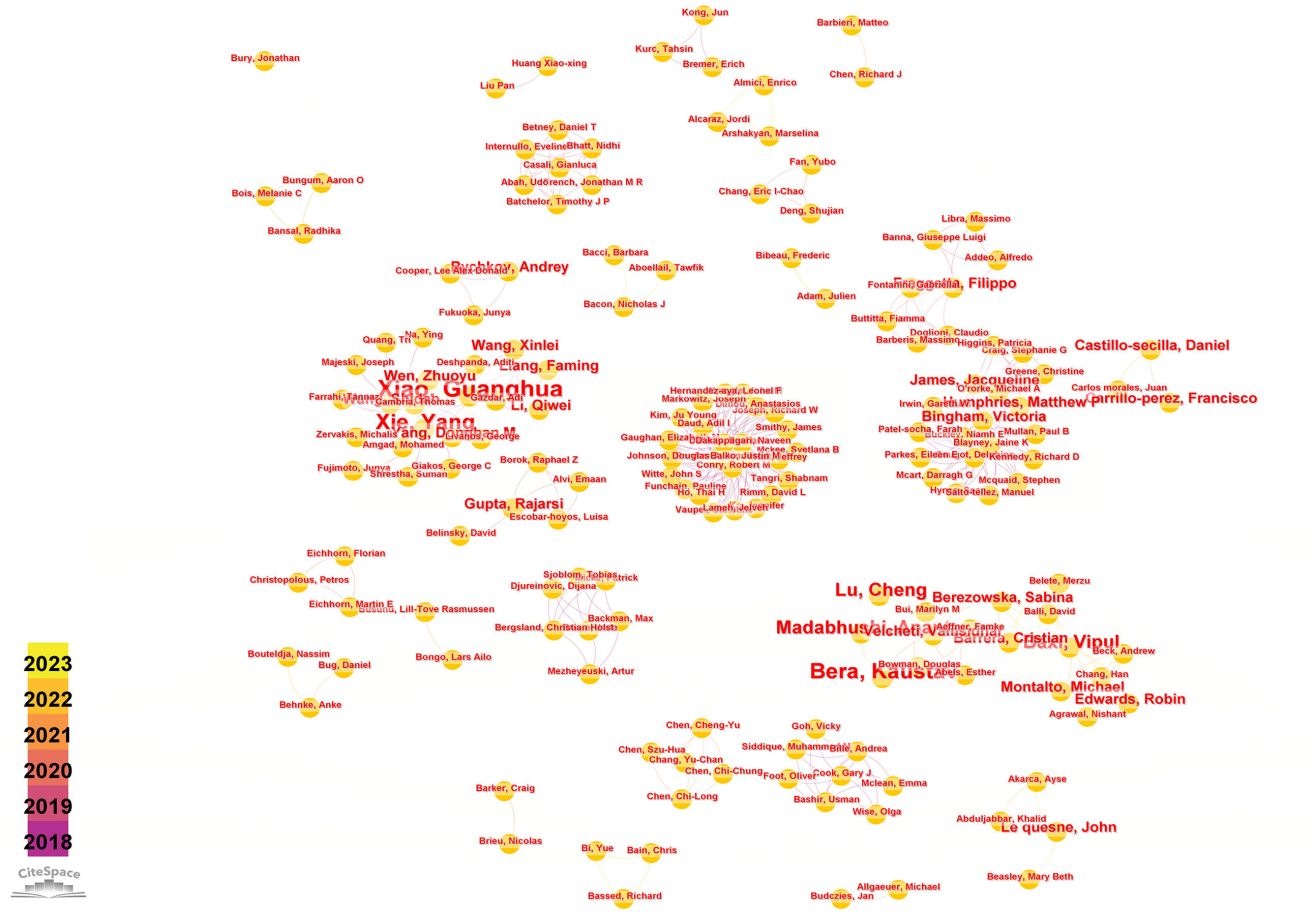
Visual map of author network.

Running the CiteSpace software, author analysis resulted in a knowledge graph with 200 nodes and 383 edges (**Figure 4**). Each circular node represents an author, and the connections between nodes represent collaborative relationships between authors. The thickness of the connections reflects the degree of collaboration. Different colors of nodes represent different time periods. Conducting a co-occurrence analysis on the author team collaboration network based on the literature retrieved from WoSCC, **Table 3** is presented, listing the top 5 authors in terms of publication output along with their affiliated institutions in this research field.

**Table 3:** Top 5 authors in terms of publication volume.

Rank	Author	Institution	Country	Number
1	Xiao, Guanghua	University of Texas Southwestern Medical Center	USA	5
2	Xie, Yang	University of Texas Southwestern Medical Center Clinic Science	USA	4
3	Bera, Kaustav	CASE WESTERN RESERVE University	USA	4
4	Baxi, Vipul	BRISTOL MYERS SQUIBB	USA	3
5	Lu, Cheng	University of ALBERTA	Canada	3

### 3.5 Co-occurrence analysis of keywords

**Figure 5:**
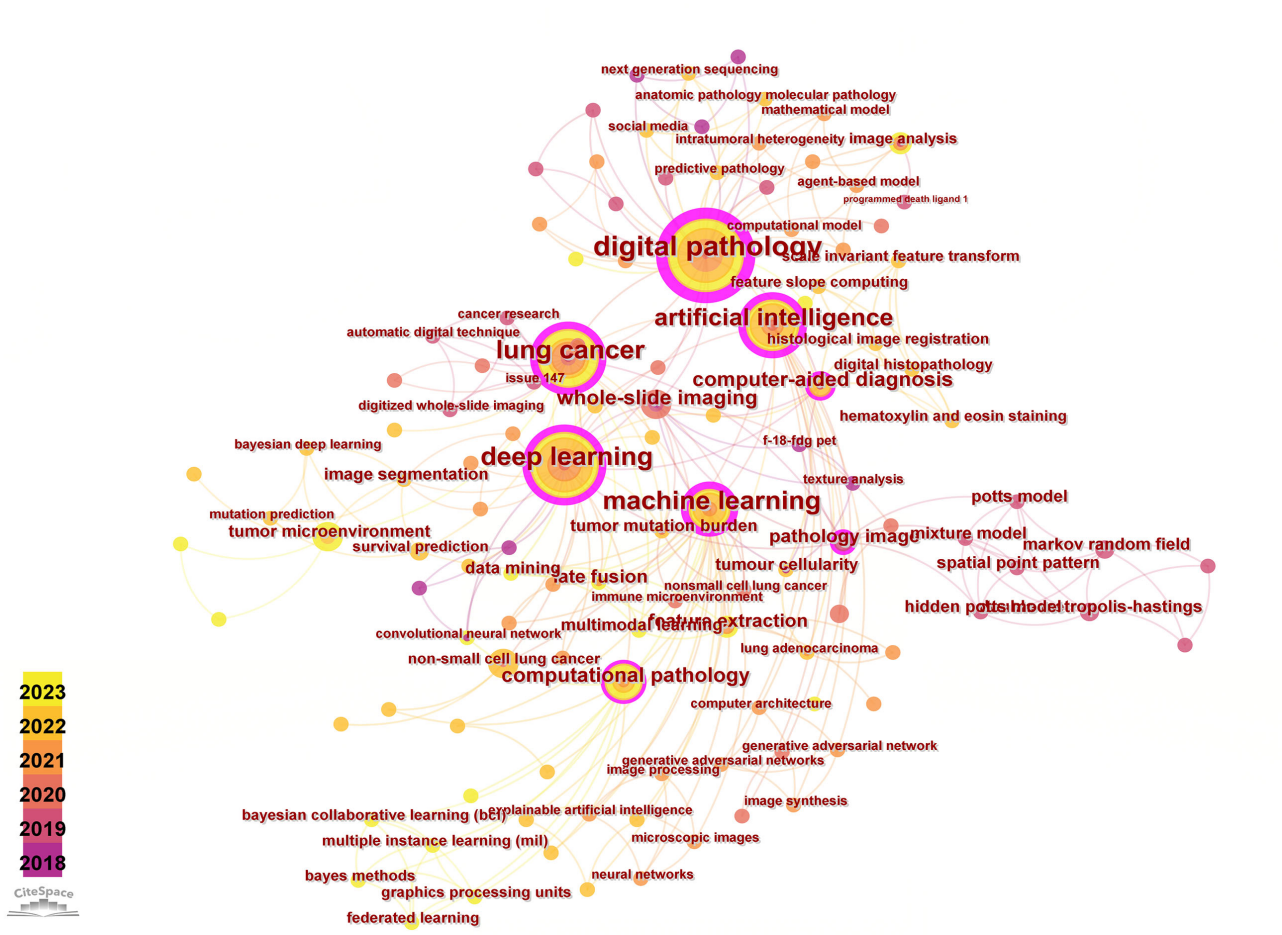
Visual map of author keywords.

**Figure 6:**
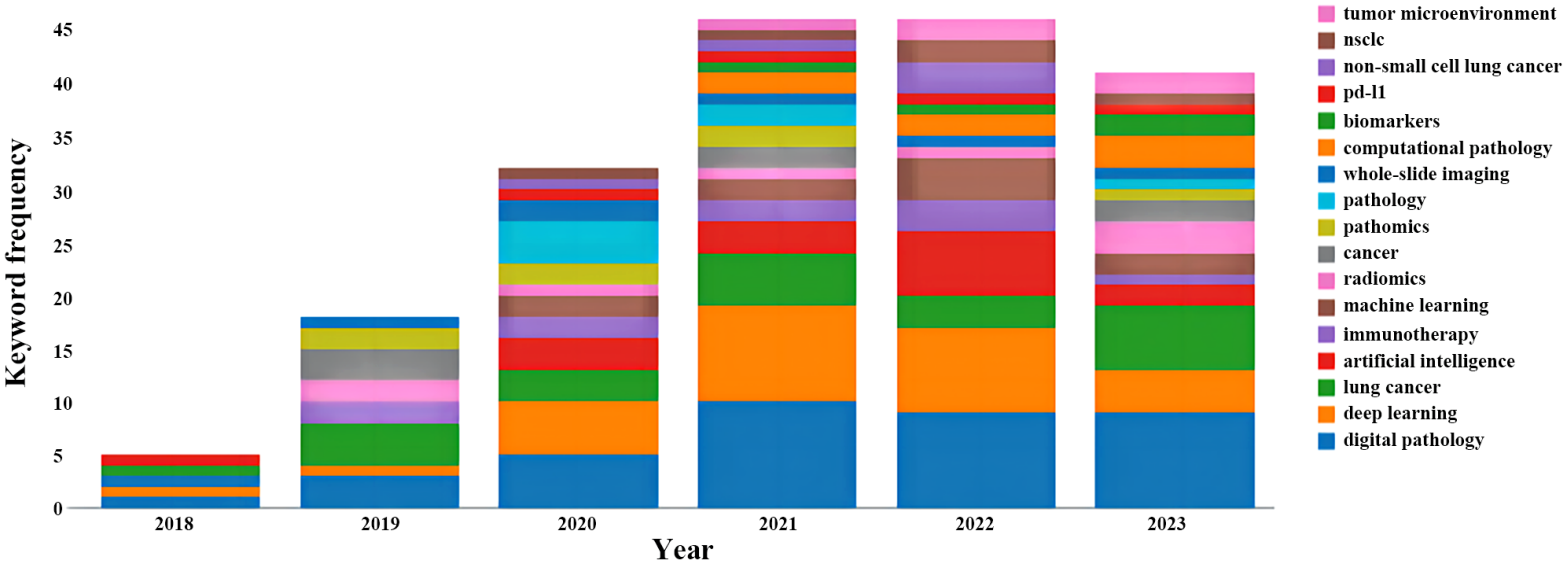
Variation in the number of keywords.

Keyword-related analysis, as manifested in the visualization of co-occurrence patterns, is crucial for delineating the research hotspots and frontiers within a given domain. Running the CiteSpace software with author keywords as node types, a co-occurrence network of keywords with 159 nodes and 334 edges was generated (**Figure 5**). After removing redundant terms that overlap with the search strategy, an analysis of the co-occurrence frequency and centrality values of keywords in this field (**Table 4**) reveals that the prominent keywords include: deep learning, artificial intelligence (AI), computer-aided diagnosis, tumor microenvironment, feature extraction, image analysis, tumor mutation burden, survival prediction, markov random field, mixture model. Furthermore, **Figure 6** illustrates the temporal frequency changes of different keywords over time. It highlights the research focal points in the past few years related to the application of AI-based pathomics in the diagnosis and treatment of lung cancer. These themes reflect the proactive role of pathomics in aiding diagnosis, classification, predicting treatment efficacy, risk assessment, exploring emerging biomarkers, and analyzing gene expression levels in the context of lung cancer diagnosis and treatment.

**Table 4:** High frequency and centrality keywords.

Rank	Keywords	Frequency	Centrality
1	deep learning	27	0.33
2	artificial intelligence	14	0.29
3	machine learning	10	0.22
4	computer-aided diagnosis	3	0.11
5	tumor microenvironment	4	0.07
6	feature extraction	3	0.04
7	image analysis	3	0.04
8	late fusion	2	0.02
9	tumor mutation burden	2	0.02
10	survival prediction	2	0.02

### 3.6 Keyword cluster analysis

Keyword cluster analysis involves utilizing the log-likelihood rate (LLR) method to analyze the connection relationships among significant keyword nodes. This method reflects the hot topics within the research domain, with closely connected keywords in a cluster indicating higher research intensity. Larger node values within a cluster signify greater research interest. By examining these clusters, it is possible to predict the developmental patterns and emerging trends in the research field (9).

**Figure 7:**
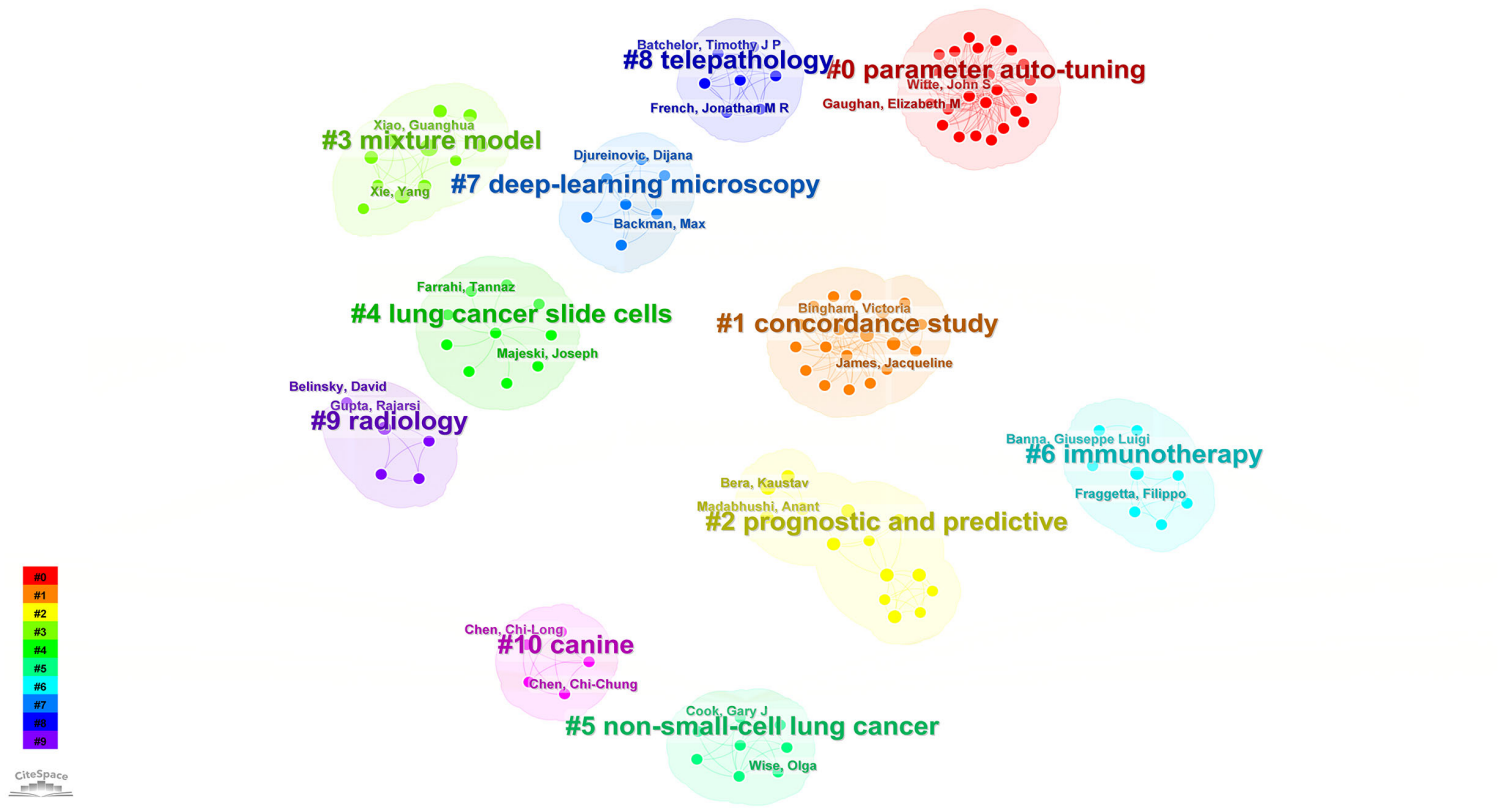
Visual map of author-generated keywords network.

**Figure 8:**
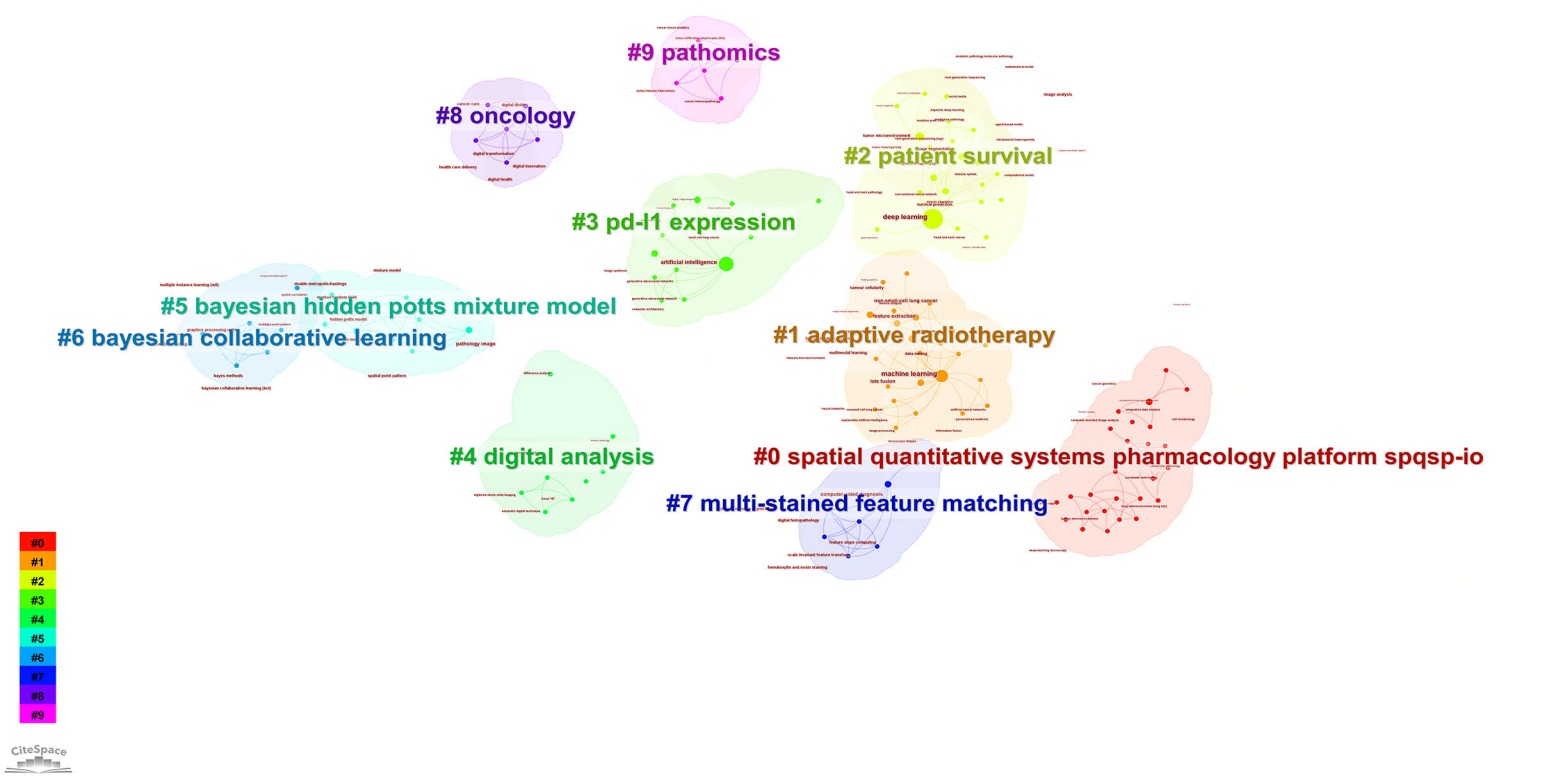
Visual map of title keywords network.

**Figure 9:**
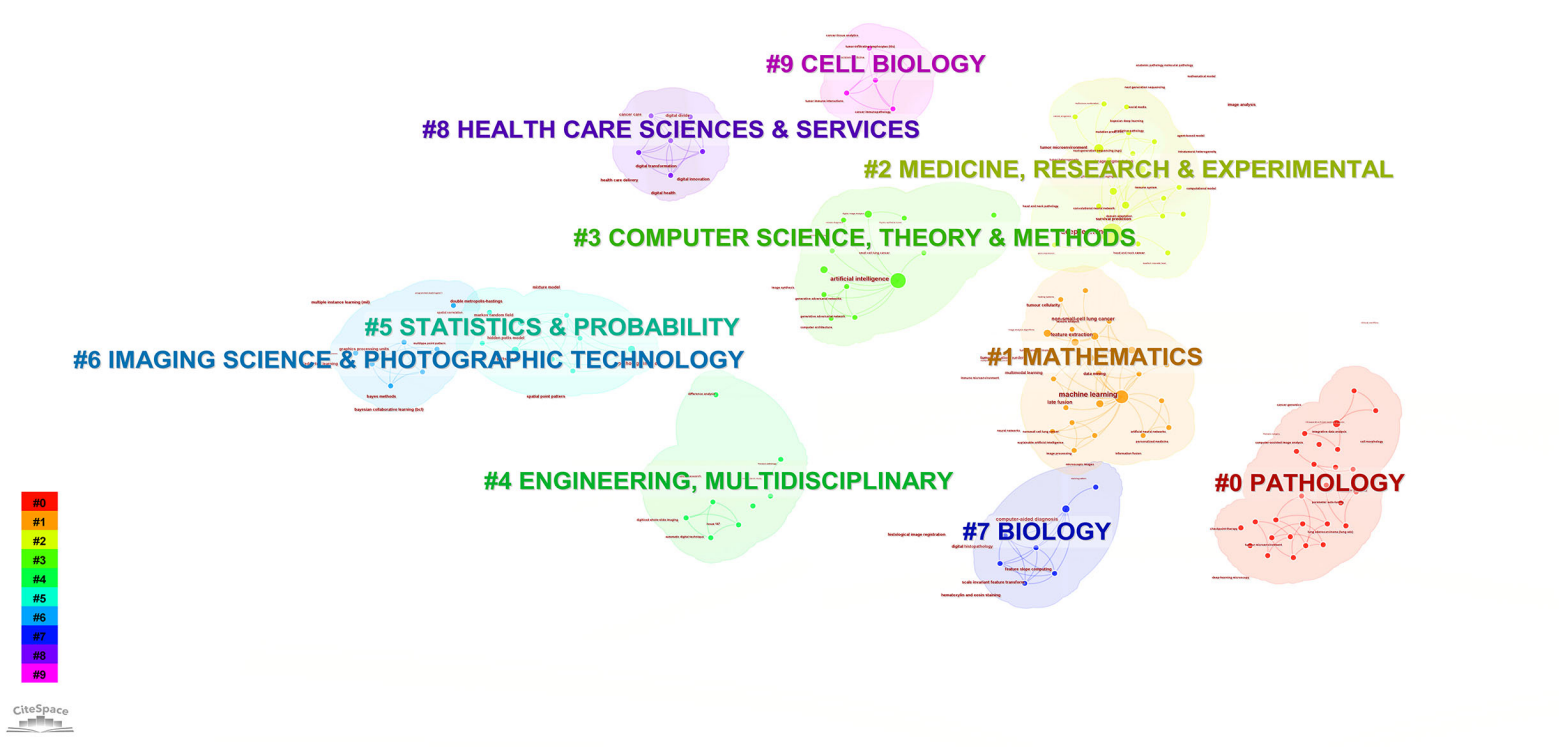
Visual map of subject categories keywords network.

**Figure 10:**
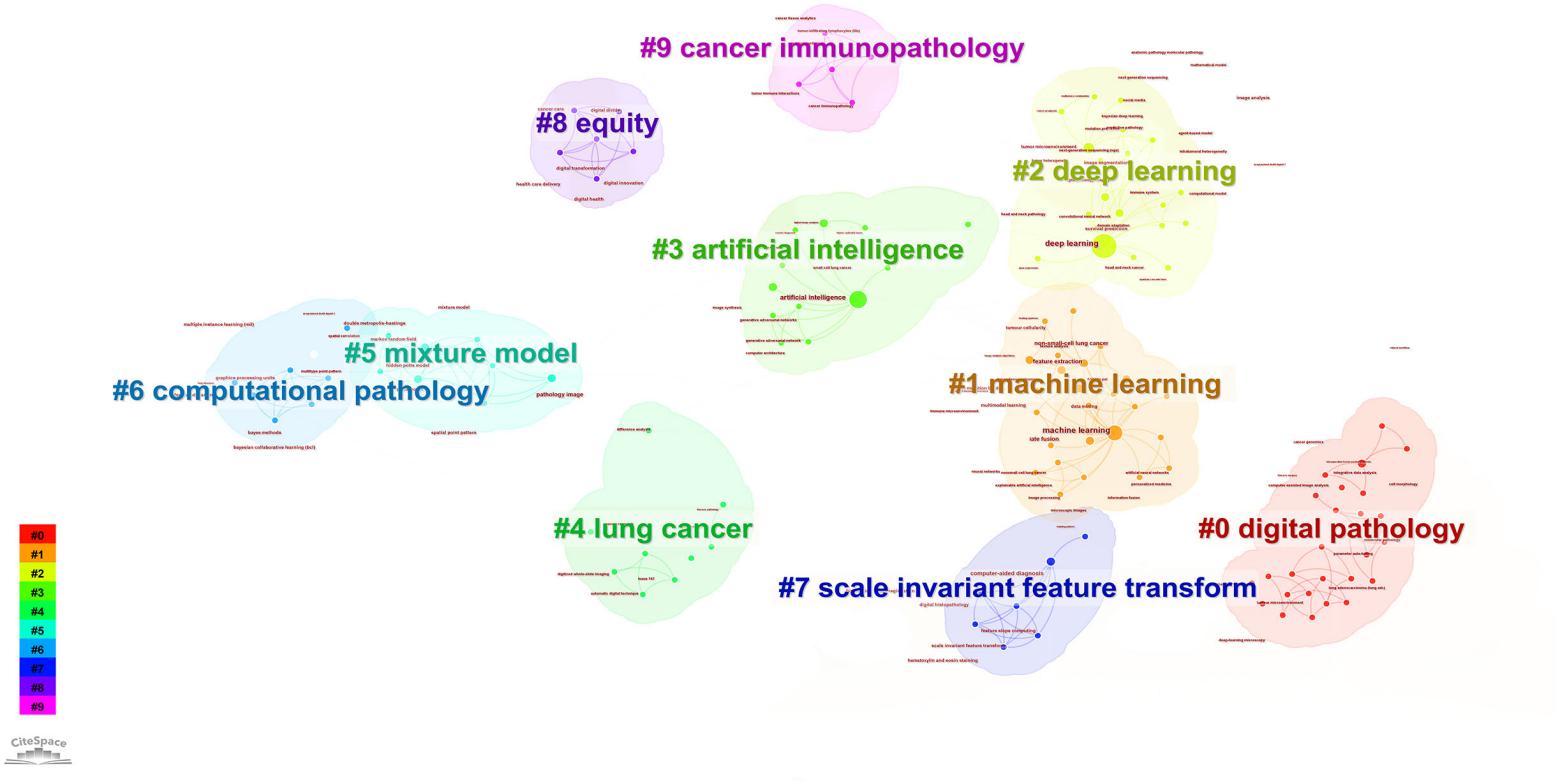
Visual map of keywords network.

According to the keyword cluster analysis (**Figure 7**), researchers’ studies are concentrated in the following 10 key areas: #0 parameter auto-tuning; #1 concordance study; #2 prognostic and predictive; #3 mixture model; #4 lung cancer slide cells; #5 non-small-cell lung cancer; #6 immunotherapy; #7 deep-learning microscopy; #8 telepathology; #9 radiology. By employing the clustering algorithm within CiteSpace software to organize title terms and visualize them (**Figure 8**), a clear sequential pattern emerges, encompassing: #0 spatial quantitative systems pharmacology platform spqsp-io; #1 adaptive radiotherapy; #2 patient survival; #3 pd-11 expression; #4 digital analysis; #5 Bayesian hidden Potts mixture model; #6 bayesian collaborative learning; #7 multi-stained feature matching; #8 oncology; #9 pathomics; Utilizing the clustering algorithm in CiteSpace to group subject categories and create a visual representation (**Figure 9**), a sequential progression of clusters is discernible, including: #0 Pathology; #1 Mathematics; #2 Medicine, Research & Experimental; #3 Computer Science, Theory & Methods; #4 Engineering, Multidisciplinary; #5 Statistics & Probability; #6 Imaging Science & Photographic Technology; #7 Biology; #8 Health Care Sciences Services; #9 Cell Biology. Employing the clustering algorithm for keywords and generating a graphical display (**Figure 10**), a sequential evolution of clusters is evident, incorporating: #0 digital pathology; #1 machine learning; #2 deep learning; #3 artificial intelligence; #4 lung cancer; # 5mixture model; #6 computational pathology; #7 scale invariant feature transform; #8 equity; #9 cancer immunopathology.

Each section is divided into 10 clustering modules, partial clustering blocks overlap with each other, suggesting close connections between these research areas. In addition to the research retrieval terms, other clusters demonstrate that pathomics in lung cancer research spans various fields, including medical experimental research, computer science, cell biology, statistics, and mathematics. Through advanced methods such as AI and machine learning, pathomics involves in-depth digital analysis of tumor tissues and the tumor microenvironment based on patients’ pathological tissue sections. It aims to construct hybrid models, identify a multitude of pathological features, conduct precise assessments, and predict tumor-related indicators, including programmed death-ligand 1 Tumor cell Proportion Score (PD-L1 TPS). The goal is to assist in personalized diagnosis and treatment for patients and contribute to clinical decision-making by leveraging the synergies between AI and clinical medicine.

### 3.7 Cited references

A total of 426 relevant articles were retrieved from WoSCC, accumulating a total of 10,174 citations. The average number of citations per article is 24. The top 10 most cited articles are listed in **Table 5**.

**Table 5:** The top 10 cited articles.

Rank	Author	Year	Total Citations	Title
1	Pucci, Carlotta;	2019	343	Innovative approaches for cancer treatment: current perspectives and new challenges (10)
2	Lu, Ming Y;	2021	278	Data-efficient and weakly supervised computational pathology on whole-slide images (11)
3	Khosravi, Pegah;	2018	180	Deep Convolutional Neural Networks Enable Discrimination of Heterogeneous Digital Pathology Images (12)
4	Acs, B.;	2020	136	Artificial intelligence as the next step toward precision pathology (13)
5	Maibach, Fabienne;	2020	118	Tumor-Infiltrating Lymphocytes and Their Prognostic Value in Cutaneous Melanoma (14)
6	Mezheyeuski, Artur;	2018	114	Multispectral imaging for quantitative and compartment-specific immune infiltrates reveals distinct immune profiles that classify lung cancer patients (15)
7	Wang, Shidan;	2019	103	Artificial Intelligence in Lung Cancer Pathology Image Analysis (16)
8	Johnson, Douglas B.;	2018	91	Quantitative Spatial Profiling of PD-1/PD-L1 Interaction and HLA-DR/IDO-1 Predicts Improved Outcomes of Anti-PD-1 Therapies in Metastatic Melanoma (17)
9	Baxi, Vipul;	2022	80	Digital pathology and artificial intelligence in translational medicine and clinical practice (18)
10	Saw, Stephanie P. L.;	2021	56	Revisiting neoadjuvant therapy in non-small-cell lung cancer (19)

## 4 Discussion

Pathomics is an innovative interdisciplinary field that combines digital pathology and AI. The rise of digital pathology has enabled the scanning of whole tissue slides, based on the fundamental principle of digitizing whole-slide images (WSI) using state-of-the-art whole-slide scanners. This technology can convert standard Hematoxylin-Eosin (H&E) staining glass slides into a digital format (WSI) (20). This allows for detailed spatial exploration of the entire tumor heterogeneity and its most invasive elements. It automatically extracts and classifies histological features, transforming this information into binary data. Finally, the extracted features are processed through sophisticated computer algorithms to perform tasks such as cancer classification and outcome prediction (21). Computational analysis of digitized histological slides through pathomics can extract valuable information. Some research primarily focuses on predicting the prognosis of lung cancer (22), including improving clinical decisions for cancer immunotherapy and exploring biomarkers related to potential benefits from ICIs, such as microsatellite instability (MSI), PD-L1 TPS, and inflammatory genes, among others (23). Another significant research area involves the integration of pathomics with multiple omics disciplines to explore the classification of lung cancer and other related aspects. Alvarez-Jimenez C et al. demonstrated the potential existence of cross-scale correlations between pathomics and CT imaging, which could be used to identify relevant imaging and histopathological features (24).

The escalating demand for personalized cancer treatment necessitates more precise biomarker assessments and quantitative tissue pathology for accurate cancer diagnosis. Pathologists must be equipped with new methodologies and tools to enhance diagnostic sensitivity and specificity, ultimately contributing to more informed and improved treatment decisions (13). Recently, significant success has been achieved in the analysis of medical images using AI due to the rapid advancement of “deep learning” algorithms (16).

Recent breakthroughs in AI hold the promise of significantly changing the way we diagnose and stratify cancer in pathology. Deep learning technology represents a milestone in this transformation, with numerous deep learning architectures applied to pathology-focused research. Various modeling objectives have been pursued, and recent studies demonstrate the application of deep learning in pathology aiming to predict conventional diagnostic features used in pathology practice (such as distinguishing between diseases and normal tissues, defining tumor grades, and differentiating cancer types), leading to new insights into diseases (25, 26).

Deep learning encompasses various types of deep neural networks, and its application has achieved several breakthroughs in addressing current key challenges in pathology (27). Convolutional Neural Networks (CNN) are the most commonly used in the analysis of pathological images (28, 29). A standard CNN consists of an input layer, task-specific output layer, and multiple hidden layers. Each hidden layer is composed of numerous convolutional filters (parameters), which apply the same local transformation at different positions in their input images (30). Due to the shared parameters when applied locally in the image, effective parameterization of the CNN model is achieved. The typical implementation of CNN models offers a degree of translation invariance, allowing detected objects or patterns to appear at any position within the image. Pooling layers are often included between convolutional layers to down-sample the intermediate outputs (feature maps) of the convolution function. Following the convolutional layers are fully connected layers, which flatten the output of the convolutional layers and generate the final representation for the input-output layers (30, 31). Each neuron in a CNN calculates its output by applying a weight vector and bias (parameters) to the input values from the previous layer. The optimization (training) of the CNN model involves iteratively adjusting these biases and weights to minimize the loss function. One advantage of CNNs over other image classification algorithms is their suitability for end-to-end learning (32). Another major advantage of CNNs is their flexibility and efficiency in learning patterns from image data. Currently, they represent state-of-the-art technology in the field of image analysis and classification, consistently outperforming earlier generations of image analysis methods (29, 32). Kao Y-S et al. conducted a study on the application of deep learning technology in histopathological tissue slices (deep pathomics) with the aim of predicting the response of stage III NSCLC to treatment (33). They assessed 35 digitalized tissue slices (biopsy or surgical specimens) from patients with stage IIIA or IIIB NSCLC. Based on the reduction in target volume observed in weekly CT scans during chemoradiotherapy, patients were categorized as responders (12/35, 34.7%) and non-responders (23/35, 65.7%). Employing a leave-two-out cross-validation method, they tested the digital tissue slices using 5 pre-trained CNNs-AlexNet, VGG, MobileNet, GoogLeNet, and ResNet, and evaluated the network performance. GoogLeNet was identified as the most effective CNN, accurately classifying 8/12 responders and 10/11 non-responders. Furthermore, deep pathomics exhibited a high level of specificity (True Negative Rate: 90.1) and considerable sensitivity (True Positive Rate: 0.75). Their data suggests that AI can surpass the capabilities of current diagnostic systems, providing additional insights beyond what is currently attainable in clinical practice.

Furthermore, there are studies attempting to apply AI to histological images with the aim of discovering novel image-based prognostic and predictive biomarkers. Cao R et al. proposed a deep learning model based on histopathological images to predict microsatellite status, achieving area under curve (AUC) of 0.88 and 0.85, respectively. It is noteworthy that this model can identify five distinct pathological imaging features, which are associated with the mutation burden in the genome, DNA damage repair-related genotypes, and the anti-tumor immune activation pathway in the transcriptome. The predictive model provides the potential for multi-omics correlations through interpretability associated with pathology, genomics, and transcriptomics phenotypes (34). Wang X et al. developed a system capable of identifying high-risk recurrence in early-stage NSCLC patients with an accuracy ranging from 75% to 82% (22). In another study, Wang S et al. characterized a group of high-risk NSCLC patients and identified image-based tumor shape features as an independent prognostic factor (35). Rakaee M et al. developed a machine learning-based scoring system for tumor-infiltrating lymphocytes (TILs) to predict the response of NSCLC to immune checkpoint inhibitor therapy (36). Additionally, Coudray N, Ocampo PS et al. applied AI to digital pathology slides to predict the presence of mutations in lung adenocarcinoma (37). In summary, the development of these advanced deep learning algorithms enhances the capability of analyzing lung cancer pathology images, assisting pathologists in challenging diagnostic tasks such as tumor identification, metastasis detection, and analysis of the tumor microenvironment.

TME is primarily composed of tumor cells, lymphocytes, stromal cells, macrophages, blood vessels, and other components. The composition of the TME varies based on the relative proportions of its different constituents, and its presence plays a crucial role in the growth and invasion of tumors.

Immune cells within the TME exhibit dual functions – on one hand, they identify and destroy tumor cells, while on the other hand, they also promote tumor growth and metastasis (38, 39). For instance, immune cells, including T cells, B cells, macrophages, and myeloid-derived suppressor cells, possess the ability to modulate the TME, thereby influencing tumor metastasis and pathological features (40, 41). Tumor Infiltrating Lymphocytes (TILs) in the TME involves a complex network of multiple cell types and cytokines and is a hallmark of immune recognition. Numerous studies have shown that activated CD8^+^ T cells are the major players involved in anti-tumor immunity, and in a subset of tumors, cancer cells inhibit the activation of CD8^+^ cytotoxic T cells through the expression of ligands such as PD-L1 that bind to inhibitory checkpoints, which has been suggested to be an important mechanism of immune escape for cancer cells (42). The expression of PD-L1 on TME immune cells, including myeloid cells (macrophages, dendritic cells) and T cells, appears to correlate more with the ICI response than expression on tumor cells. However, in NSCLC clinical practice, a limitation in histologically characterizing T lymphocyte infiltration is the scarcity of tumor tissue, which has hampered insight into the role of T lymphocytes in influencing the ICI response (43). Tumor-associated macrophages can promote angiogenesis and invasion by secreting cytokines, growth factors, and proteases (44). Cancer-associated fibroblasts (CAF) are pivotal in the formation of organs and the maintenance of tissue structure and function. They also play a significant role in tumor initiation, progression, metastasis, and the development of drug resistance through their potent immunosuppressive capabilities. Activated CAF possess the capability to secrete various substances, including extracellular matrix and vascular endothelial growth factor (VEGF), contributing to the complexity of the TME (45, 46). The markers associated with CAF are predominantly linked to T cell immunosuppression, inhibiting the functions of CD8^+^ T cells and natural killer cells, particularly by secreting various chemokines and cytokines, notably interleukin-6 (IL-6), which leads to suboptimal clinical treatment outcomes. As research into the effects of CAF and the TME on immune cells and the efficacy of cancer immunotherapy advances, scientists can potentially develop novel compounds targeting these mechanisms, thereby offering innovative strategies for immunotherapy (47). It is noteworthy that research indicates a significant impact of the TME on the survival benefits of immunotherapy (48). The presence of immune cells in the TME, including the percentage of CD8^+^ T cells, can serve as a predictive factor for the effectiveness of immunotherapy (49). The extracellular matrix can influence the mechanisms of tumorigenesis by affecting cell growth, metastasis, and immune evasion through the activation of signaling pathways. Additionally, tumor cells have the capability to release various growth factors, such as tumor growth factor, endothelial growth factor, and VEGF, contributing to the promotion of new blood vessel development (50). Angiogenesis is crucial for providing nutrients and oxygen to tumor cells, ultimately playing a critical role in tumor growth.

Therefore, TME plays a crucial role in tumor growth and metastasis. A comprehensive understanding of TME formation, investigating the interplay between immune cells and tumors, and exploring various genetic variations represent the future directions of TME research (51, 52). Additionally, selecting targeted therapeutic strategies based on TME subtypes can enhance the effectiveness of cancer treatment. To further emphasize this point, computer-assisted automatic detection of tumor cells in lymph nodes can significantly reduce the false-negative rate, thereby facilitating earlier detection and treatment of lung cancer, improving the accuracy of TNM staging, accelerating the examination process, and reducing the workload of pathologists. Moreover, tumor spread through air spaces (STAS) has been identified as an important clinical factor associated with tumor recurrence and poor prognosis in patient survival. The identification and quantification of STAS require experienced pathologists to perform detailed examinations of entire tissue sections. Therefore, pathological image analysis tools that rapidly and accurately identifies STAS would be useful for pathologists (16). Quantitative characterization of TME and accurate prediction and classification of important TME components are essential for targeted tumor therapy and prognosis assessment (53), necessitating advanced data processing and analysis approaches.

Quantitative characterization of TME involves a crucial step of segmenting different types of tissue substructures and cells from pathological images. This segmentation forms the foundation for various image analysis tasks, including cellular composition, spatial organization, and morphology specific to substructures. Previous studies in oncology primarily focused on tumor cells, overlooking the pivotal role of TME in the initiation and progression of cancer. The TME of lung cancer is primarily composed of tumor cells, lymphocytes, stromal cells, macrophages, blood vessels, and other components. Studies in lung cancer have indicated that TILs are positive prognostic factors, while angiogenesis is negatively associated with survival outcomes. The role of stromal cells in prognosis is complex. Traditional image processing methods encompass feature definition, feature extraction or segmentation. These techniques have been employed to segment lymphocytes and analyze the spatial organization of TILs and stromal cells within the TME (54). Research associated with the quantitative characterization of TME has the potential to predict treatment outcomes and provides insights for the development of targeted therapeutic strategies. Innovative studies in immunotherapy, in particular, heavily rely on understanding the interactions among various components within the TME and the mechanisms of immune evasion.

Accurate characterization of specific structures and features of TME is crucial for evaluating tumor prognosis (55), enhancing clinical decisions, and advancing precision medicine. Radiomics can unveil the heterogeneity of tumor cells and TME, while genomics and pathomics explore the biological significance of imaging histological features. The integration of these three approaches contributes to a comprehensive understanding and decoding of TME characteristics in tumors, facilitating prognostic predictions (56). The interconnection between radiomics, pathomics, and genomics contributes to establishing and deepening our understanding of cancer biology and imaging features. Concurrently, powerful machine learning techniques can decipher the complex interactions between tumors and cancer treatments. The integration of machine learning technologies with digital imaging and novel methods for assessing TME at the molecular level significantly enhances our comprehension of TME and cancer prognosis assessment. Vanguri RS et al. employed machine learning to integrate multimodal features into a risk prediction model (57). By combining radiological, histopathological, and genomic features, they assessed the predictive capability of immunotherapy response in NSCLC. Their study revealed that the AUC value of the multimodal model was 0.80, surpassing any single variable. These findings establish a quantitative foundation for enhancing the accuracy of predicting immunotherapy response in NSCLC patients through the integration of multimodal features and machine learning.

Simultaneously, the quantitative characterization of TME in lung cancer poses certain challenges, including the following aspects: (1) Complexity and heterogeneity of lung cancer TME composition: In addition to the mentioned cell types, other structures such as bronchi, cartilage, and pleura often appear in pathological sections of the lung. This complexity and heterogeneity make segmentation and traditional feature definition challenging. (2) Cellular spatial organization (e.g., spatial distribution and interactions of different cell types): While playing a crucial role in TME, it is more challenging to capture than simply providing the quantity or ratio of different cell types. Current research mainly focuses on the proportion of different cell types, overlooking the intricate cellular spatial organization, which may result in limited and contradictory outcomes regarding the roles of different cell types in the TME. (3) For H&E-stained glass slides, there can be significant color variations based on staining conditions and the time gap between slide preparation and scanning. Traditional image processing methods based on manual feature extraction struggle to overcome these obstacles. (4) Multi-omics studies face the high dimensionality and heterogeneity of data, and integrating quantitative measurements of multi-modal data for prognosis prediction is a highly challenging task. In summary, pathomics, as a nascent research methodology, is presently undergoing preliminary investigation. Future studies utilizing extensive multi-omics datasets have the potential to advance the formulation of sophisticated integration strategies. These strategies would facilitate a more exhaustive evaluation, characterization, and elucidation of TME (58). Consequently, this advancement will yield profound insights into the imaging characteristics and the pathophysiological and biological underpinnings of tumor pathology.

In recent years, amidst the high incidence and mortality rates of lung cancer, the selection and implementation of treatment plans for advanced-stage lung cancer patients, as well as the creation of more precise platforms for predicting treatment responses, continue to face challenges. Pathomics not only synergizes with traditional pathological semantic information and clinical data to discover disease patterns but also interacts and integrates with various omics information, leveraging the unique advantages of each omics discipline. The development of these interdisciplinary approaches not only aids in identifying subtle lesions that may escape the naked eye and uncovering disease patterns beyond subjective judgment but also facilitates relatively objective and accurate assistance in disease screening, diagnosis, differential diagnosis, and prognosis assessment. Furthermore, it contributes to saving human and material resources, optimizing the utilization of limited medical resources to the maximum extent, and, on a broader scale, promoting the development of the personalized immune intervention.

## 5 Conclusion

In conclusion, this study systematically analyzed the literature on pathomics in the management of lung cancer indexed within the WoSCC. It offers an initial overview of recent research trends and forecasts potential hotspots and frontiers for future inquiry, aiming to provide valuable insights and references for scholars and researchers involved in personalized immunotherapy efficacy and prognosis for lung cancer.

## Data availability statement

The original contributions presented in the study are included in the article/supplementary material. Further inquiries can be directed to the corresponding author.

## Author contributions

LY: Validation, Visualization, Writing – original draft, Writing – review & editing. ZS: Validation, Writing – original draft, Writing – review & editing. YS: Data curation, Validation, Writing – original draft, Writing – review & editing. JZ: Data curation, Software, Validation, Visualization, Writing – review & editing. QW: Data curation, Software, Validation, Visualization, Writing – review & editing. YL: Data curation, Visualization, Writing – review & editing. HS: Supervision, Validation, Writing – review & editing.
